# Pulmonary Exacerbation of Undiagnosed Toxocariasis in Intensively-Treated High-Risk Neuroblastoma Patients

**DOI:** 10.3390/children7100169

**Published:** 2020-10-05

**Authors:** Szymon Janczar, Monika Bulas, Justyna Walenciak, Dobromila Baranska, Marek Ussowicz, Wojciech Młynarski, Beata Zalewska-Szewczyk

**Affiliations:** 1 Department of Pediatrics, Oncology and Hematology, Medical University of Lodz, Sporna 36/50 St., 91-738 Lodz, Poland; mbulas@wp.pl (M.B.); justyna.walenciak@umed.lodz.pl (J.W.); wojciech.mlynarski@umed.lodz.pl (W.M.); beata.zalewska-szewczyk@umed.lodz.pl (B.Z.-S.); 2 Department of Diagnostic Imaging, Polish Mother’s Memorial Hospital Research Institute, 91-738 Lodz, Poland; dobaranska@gmail.com; 3 Department of Pediatric Bone Marrow Transplantation, Oncology and Hematology, Wroclaw Medical University, 50-556 Wroclaw, Poland; ussowicz@o2.pl

**Keywords:** neuroblastoma, toxocariasis, autologous stem cell transplantation, Toxocara

## Abstract

Toxocariasis is one of the most common zoonoses, with high seroprevalence in apparently healthy individuals. Neuroblastoma is an aggressive childhood cancer. The cure rates are improving due to dose-dense chemotherapy, progress in surgical practice, myeloablative therapy with autologous stem cell transplantation, and recently, anti-GD2 immunotherapy. This is associated with a burden of complications, some of which are relatively specific for neuroblastoma treatment. Based on previous reports of *Toxocara canis* infection in high-risk neuroblastoma patients and cases of pulmonary exacerbation from our center in this disease, we propose that toxocariasis is a specific complication of intensive pediatric cancer treatment and advocate for active surveillance.

## 1. Introduction

Here, we report on two children with undiagnosed *Toxocara canis* infestation that developed pulmonary symptoms during intensive high-risk neuroblastoma treatment. Toxocariasis is one of the most common zoonoses in the world, and its IgG seroprevalence might be roughly estimated at up to 20% worldwide and up to 10% in Europe, with wide variance between studies and regions [1,2]. Toxocariasis is caused by infestation of humans by ascarid larvae belonging to the Toxocara genus from dogs and cats, and has varied clinical manifestations including systemic and compartmentalized syndromes as well as asymptomatic cases. Pulmonary involvement is usually a component of classic visceral larva migrans syndrome, which is associated with high eosinophilia, hepatosplenomegaly, fever, and hyper-gammaglobulinemia [3,4]. The descriptions of toxocariasis in cancer patients are scarce. Disseminated neuroblastoma is one of the most aggressive childhood cancers and is still associated with high mortality. While historically the cure rates were below 5%, they have gradually improved due to treatment intensification including dose-dense induction chemotherapy, progress in surgical techniques, the introduction of meyloablative chemotherapy (MAT) with autologous hematopoietic stem cell transplantation (autoSCT) around 30 years ago, and more recently, anti-GD2 immunotherapy. Still, serious complications are common and significant in the course of this disease, and some of them have a degree of specificity for high-risk neuroblastoma treatment. This can be exemplified by a high frequency and severity of venoocclusive disease of the liver (VOD), which is believed to be associated with the disease, but more likely with the conditioning prior to autoSCT, in particular, BuMEL [5,6,7] (while important and frequently unique, complications of neuroblastoma immunotherapy are not a subject of this work). Here, based on previous reports of *Toxocara canis* infections in post-transplant neuroblastoma patients [4] and two cases of pulmonary toxocariasis exacerbation from our center, we propose that pulmonary toxocariasis should be considered a specific complication of intensive pediatric cancer therapy such as dose-dense chemotherapy and MAT/SCT used in neuroblastoma treatment. 

## 2. Results

**Figure 1:**
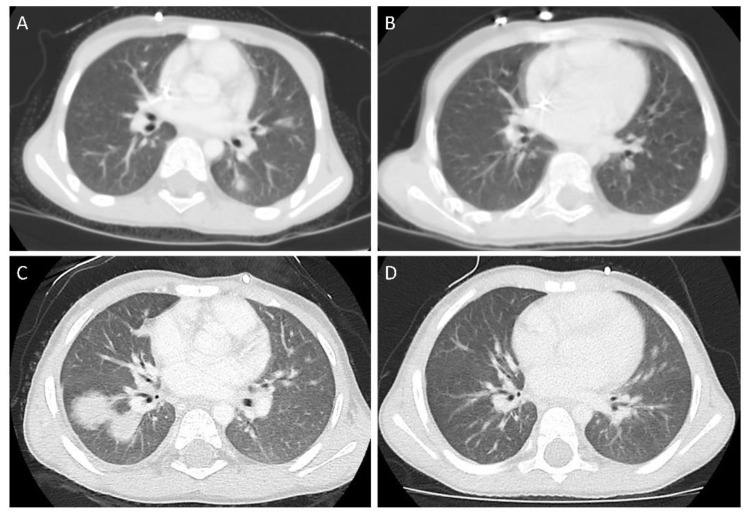
Pulmonary lesion pre- (**A**,**C**) and post- (**B**,**D**) antiparasitic treatment in Patient 1 (A,B) and Patient 2 (C,D). A: left lung, inferior lobe, segment 6. C: right lung, inferior lobe, segments 6, 8, 10.

In our center (Department of Pediatrics, Oncology and Hematology, Medical University of Lodz, Poland), the patients with high-risk neuroblastoma are treated according to the subsequent versions of European HR-NBL/SIOPEN (High-Risk Neuroblastoma /International Society of Paediatric Oncology Europe Neuroblastoma) Protocols. In the decade between 2010–2019, we treated, in total, 21 patients with high-risk neuroblastoma. For MAT/SCT (meyloablative chemotherapy /stem cell transplantation), the patients are referred to the Department of Pediatric Bone Marrow Transplantation, Oncology, and Hematology, Wroclaw Medical University, Wroclaw, Poland, which is the most active pediatric BMT (bone marrow transplantation) unit in our country. In two male patients, separated by an interval of 3.5 years, both coming from rural areas with history of pet exposure, in planned imaging studies performed in the first case after induction of rapid COJEC (cisplatin, vincristine, carboplatin, etoposide, and cyclophosphamide) therapy and before tumor resection (Patient 1), and in the second case (Patient 2) after MAT/SCT (42 days post SCT), we found focal lesions in the lungs. Pulmonary recurrence/metastases were strongly suspected, and the representative computed tomography (CT) scans are presented in Figure 1. Both patients were apparently asymptomatic at that time. Surprisingly, in both cases, scintigraphy was negative for pathological 123I mIBG uptake in the chest, suggesting no active disease in the lungs. In one case (Patient 2), we proceeded to surgical biopsy, with the histopathological examination demonstrating no neoplastic tissue, whereas in the other patient (Patient 1), the biopsy was not plausible anatomically. As in both patients there was a marked eosinophilia in the peripheral blood, and there are descriptions of toxocariasis in children treated for high-risk neuroblastoma [4], we also performed serological studies for toxocariasis, and the serology was positive in both patients. The details of treatment and diagnostics of both patients are summarized in Table 1. We initiated toxocariasis treatment with albendazole in both patients with variable success. Patient 1 had features of relapsing infestation. He received two courses of albendazole prior to MAT/SCT and was free from signs of toxocariasis, though with positive serology, while MAT was initiated. He was not tested for *T. canis* and there was no hypereosinophilia during the hospitalization post SCT in the BMT Unit. Following full hematopoietic reconstitution, while there were no abnormal radiological findings in the lungs, we observed a relapse of hypereosinophilia (reaching a maximal value of 9.500 /µL) and skin rash around four weeks post SCT. He was again treated with albendazole, with no improvement in laboratory markers of toxocariasis, and eventually the patient was switched to diethylcarbamazine (especially in light of scheduled potentially associated with adverse events in anti-GD2 immunotherapy). His eosinophil count was finally normal following two courses of diethylcarbamazine, and there were no features of toxocariasis recurrence later during treatment and follow-up. Patient 2 responded favorably to the second course of albendazole following relapse of hypereosinophilia two months post first albendazole therapy. Figure 1 demonstrates the lesions found in initial imaging (lung CT) after autoSCT and the resolution of changes following anti-parasitic treatment. In both patients, we proceeded with further treatment according to the HR NBL/SIOPEN Protocols, as presented in Table 1.

## 3. Discussion

Toxocarosis is one of the most common zoonoses in the world. In Poland, between 1994 and 2005, there were about 16.6–75.6% of seropositive results in various studies of individuals suspected of being infected with Toxocara, but nationwide IgG-seroprevalence is unknown [8]. The therapy is always recommended due to the risk of complications caused by accidental larva migration to the eyes or central nervous system. Pulmonary toxocariasis is a serious complication, and is usually treated with albendazole and corticosteroids [9]. Here, we propose that toxocariasis reactivation, and particularly pulmonary involvement, should be added to a list of potential complications of pediatric cancer therapy such as in patients with high-risk neuroblastoma treated with dose-dense chemotherapy or MAT/SCT in the setting of the HR NBL/SIOPEN Protocol, which is a perfect example of multimodal pediatric oncology efforts. Such multimodal treatment is associated with a wide catalogue of complications of varied frequency and severity. These include hematological toxicities, infections, organ toxicities, capillary leak syndrome, gastrointestinal syndromes, and neurotoxicity [5,6,7,10]. In fact, the benefits of MAT/SCT in neuroblastoma in the era of immunotherapy should be re-examined [11,12].

Here, we point to the fact that there is a relative paucity of reports of toxocariasis in the setting of neoplasia. Apart from our patients and another series of neuroblastoma patients from Poland, the reported cases or case series include Wilms tumor [13,14,15], lung [16], bowel cancer [17,18], osteosarcoma [19], retinoblastoma [20], and mycosis fungoides [21]. Surprisingly, there are no reports of clinical Toxocara syndromes in leukemias. In general, the available published data are very limited, in particular, any studies (such as, for example, seroprevalence study) of larger cohorts of cancer patients are lacking. We reviewed the medical data of our oncohematology center and also of the BMT unit that transplanted both patients and found no other records of diagnoses of toxocariasis, however, there was no preventive surveillance in patients with no clinical suspicion. The rate of pulmonary toxocariasis in high-risk neuroblastoma patients in our center (9.5%) is only slightly lower than the rate of serologically proven toxocariasis in a previous report from Poland (14%, with one patient out of three with pulmonary involvement). To some extent, toxocariasis might be associated with conditioning schedule as both patients from our study (in Patient 2 proceeding the initial diagnosis and in Patient 1 prior to albendazole-resistant recurrence) and two out of three from the previous report received BuMEL. The cases from our report had rather mild clinical course, however, it is very difficult to exclude that complications observed, for example, post SCT/BMT obscured the symptoms of toxocariasis. Furthermore, the expression of systemic toxocariasis features might have been reduced by lack of immunocompetence and ability for inflammatory response in our patients.

In conclusion, we report that toxocariasis reactivation, and in particular pulmonary morbidity, is a potential threat in pediatric cancer patients. We advocate for active *T. canis* infection surveillance in children with cancer both for research purposes (i.e., establishing the frequency of toxocariasis) and to aid clinical decisions in the case of unexplained systemic, pulmonary, hepatic, neurological, or ophthalmic complications. Pediatric patients should be screened for zoonotic diseases that can exacerbate during intensive therapy. The regularly repeated deworming or preferably temporary household removal of pets in families with immunocompromised members should be recommended, and enforcing of local veterinary guidelines is highly advised.

## Figures and Tables

**Table 1:** The clinical, laboratory, radiological, and histopathological characteristics of both patients.

	Patient 1	Patient 2
Demographic		
Treatment commenced	2016	2019
Sex	Male	Male
Age (years)	2.5	2.5
Clinical and pathological features at diagnosis		
Primary site	Abdomen	Chest
Metastases	None	Bones, bone marrow
INSS Stage	3	4
Histology	Neuroblastoma –non otherwise specified	Neuroblastoma –undifferentiated
N-Myc status	Amplified	Non-amplified
Protocol	HR NBL/SIOPEN 1.5	HR NBL/SIOPEN 1.7
Treatment and response prior to MAT/SCT		
Induction chemotherapy	Rapid COJEC	Rapid COJEC
Stem cell harvest	Post induction, G-CSF mobilization	Post induction, G-CSF mobilization
Response post induction	Complete remission, 123I mIBG negative	Very good partial response /metastatic partial remission (residual 123I mIBG activity in the bones, chest negative)
Tumor resection	Post induction, complete	Post induction, complete
MAT /SCT		
Conditioning	Busulfan 16 × 1 mg/kg + Melfalan 140 mg/m^2^	Busulfan 16 × 1.2 mg/kg +Melfalan 140 mg/m^2^
Stem cell load	6.26 × 10^6^ CD34+/kg	5.35 × 10^6^ CD34+/kg
WBC > 1000/µL	Day +11	Day +11
ANC > 500/µL	Day +11	Day +11
Platelets > 20000/µL	Day +13	Day +35
Non-hematological complications post SCT	C. difficile infection, mucositis	Pyelonephritis, VOD, mucositis
Specific features during Toxocara infestation		
Relation to neuroblastoma treatment	Post induction and stem cell apheresis	42 days post MAT/SCT
Biopsy /histopathology	Not performed	No neoplastic cells, macrophage infiltration
Highest Eosinophil count	9.500/µL	4.100/µL
Total IgE (*N* < 60 IU/mL)	937 IU/mL	normal
Immunoglobulins (IgG, IgA, IgM)	normal	normal
Lymphocyte subpopulations	CD3 and CD19 lymphopenia	Not performed
HIV	Negative	Negative
Antiparastic treatment	Albendazole 2 courses prior to SCT and 1 post SCT (10 days each) Diethylcarbamazine 3 mg/kg 3 times daily for 21 days (2 courses)	Albendazole -2 courses (10 days each)
Follow-up		
Follow-up since diagnosis	4 years, in remission, good general condition, no features of toxocariasis	6 months, in remission (no 123I mIBG activity, no features of local recurrence, normal tumor markers), good general condition, currently undergoes immunotherapy, no features of toxocariasis

INSS: International Neuroblastoma Staging System; HR-NBL/SIOPEN: High-Risk Neuroblastoma /SIOP Europe Neuroblastoma; MAT/SCT: meyloablative chemotherapy /stem cell transplantation; COJEC: cisplatin, vincristine, carboplatin, etoposide, and cyclophosphamide; G-CSF: granulocyte-colony stimulating factor; mIBG: meta-iodobenzylguanidine; VOD: veno-occlusive disease of the liver; IU: international units.
